# Chlorophyllin Modulates Gut Microbiota and Inhibits Intestinal Inflammation to Ameliorate Hepatic Fibrosis in Mice

**DOI:** 10.3389/fphys.2018.01671

**Published:** 2018-12-04

**Authors:** Han Zheng, Yang You, Meiyun Hua, Pengfei Wu, Yu Liu, Zishuo Chen, Li Zhang, Haoche Wei, Yan Li, Mei Luo, Yilan Zeng, Yong Liu, Dong-Xia Luo, Jie Zhang, Min Feng, Richard Hu, Stephen J. Pandol, Yuan-Ping Han

**Affiliations:** ^1^Key Laboratory of Bio-Resource and Eco-Environment of Ministry of Education, The Center for Growth, Metabolism and Aging, The College of Life Sciences, Sichuan University, Chengdu, China; ^2^Chengdu Tongde Pharmaceutical Ltd., Chengdu, China; ^3^Public Health and Clinical Center of Chengdu, Chengdu, China; ^4^Nanjing Drum Tower Hospital, The Affiliated Hospital of Nanjing University Medical School, Nanjing, China; ^5^Olive View-UCL Medical Center, Los Angeles, CA, United States; ^6^Cedars-Sinai Medical Center, Los Angeles, CA, United States

**Keywords:** sodium copper chlorophyllin, liver fibrosis, intestinal tissue barrier, gut microbiota dysbiosis, NF-κB pathway

## Abstract

Liver fibrosis is an abnormal wound healing response and a common consequence of chronic liver diseases from infection or alcohol/xenobiotic exposure. At the cellular level, liver fibrosis is mediated by trans-differentiation of hepatic stellate cells (HSCs), which is driven by persistent hepatic and systemic inflammation. However, impaired enterohepatic circulation and gut dysbiosis may indirectly contribute to the liver fibrogenesis. The composition of the gut microbiota depends on diet composition and host factors. In this study, we examined chlorophyllin, derived from green pigment chlorophyll, on gut microbiota, the intestinal mucosal barrier, and liver fibrosis. BALB/c mice received carbon tetrachloride through intraperitoneal injection to induce liver fibrosis and chlorophyllin was administrated in drinking water. The effects of chlorophyllin on liver fibrosis were evaluated for (1) survival rate, (2) hepatic morphologic analysis, (3) inflammatory factors in both the small intestine and liver, and (4) gut microbiota. Our results indicate that oral administration of chlorophyllin could attenuate intestinal and hepatic inflammation and ameliorate liver fibrosis. Importantly, oral administration of chlorophyllin promptly rebalanced the gut microbiota, exhibiting down-regulation of the phylum Firmicutes and up-regulation of the phylum Bacteroidetes. *In vitro* experiments on intestinal epithelial cells showed that chlorophyllin exposure could inhibit NF-κB pathway via IKK-phosphorylation suppression. In conclusion, this study demonstrates potential application of chlorophyllin to regulate the intestinal microbiota and ameliorate hepatic fibrosis.

## Introduction

Cirrhosis is a common consequence of various types of chronic liver diseases, derived from viral infection, alcohol abuse and drug/xenobiotic exposure, bile duct obstruction, and fatty liver (steatosis). At the cellular basis, liver fibrosis us the trans-differentiation or activation of hepatic stellate cells (HSCs) (Rippe and Brenner, 2004; Friedman, 2008). As vitamin A storing cells located in the space of Disse in the liver, quiescent HSCs are the major hepatic parenchymal cells that produce the extracellular matrix (ECM) to support the normal liver cellular architecture in the form of hepatic sinusoids. Chronic hepatic necro-inflammation, derived from liver injury and infection, is a major driving force for liver fibrosis. For instance, our previous work showed that interleukin-1 as an injury signal was able to activate HSC through expressing matrix metalloproteinases (MMPs), which consequently could release transforming growth factor-beta (TGF-beta) stored in the ECM scaffolds in the space of Disse (Lu et al., 2013). Persistent inflammation and repeating cycles of infection/injury and wound healing can lead to excessive wound healing and scarring, which results in cirrhosis, while genetic depletion of the interleukin-1 receptor or neutralization of Tregs can attenuate liver fibrogenesis (Gieling et al., 2009; Zhang et al., 2016).

On the other hand, gut microbiota is critical for host health and well-being. Gut microbes assume many physiological functions for their hosts such as the production of vitamin B series, energy harvesting from undigested diet, and the promotion of host immunity and antagonizing foreign invasion. In contrast, dysbiosis, mediated via the production of endotoxin, bacterial antigenic debris (including DNA fragments), and metabolites, is detrimental for the host. It has been reported that gut bacterial translocation, measured via bacterial DNA and peptide fragments as well as enterohepatic circulation, can elicit inflammation and promote liver fibrosis (Neugebauer et al., 2008; Cano et al., 2010; Bellot et al., 2013). The enterotype of the gut microbiota is determined by host immunity and environmental factors including dietary composition. Consuming green vegetables, due to the green pigment (chlorophyll) in addition to dietary fibers and vitamins, has been suggested to impact human health and physiological functions. Chlorophyllin, derived from chlorophyll, is a major component that has been widely used as a green pigment in the food industry. A clinical trial suggested that chlorophyllin administration could reduce the effects of aflatoxin and improved liver carcinogenesis (Egner et al., 2001). In chemically induced animal models of carcinogenesis, dietary supplementation of chlorophyllin was found to protect the animal from genomic instability (Vidya Priyadarsini et al., 2012). Similarly, carcinogen induced rat forestomach carcinogenesis could be ameliorated by dietary chlorophyllin via modulation of TGF-beta signaling and downstream target genes associated with cell proliferation, apoptosis evasion, angiogenesis, invasion, and metastasis (Thiyagarajan et al., 2014). Whether dietary supplementation with chlorophyllin can modulate gut microbiota and impact liver injury and cirrhosis remains completely unknown. In this study, we measured such effects and found that chlorophyllin can promptly modulate the gut microbiota and reduce hepatic inflammation to relieve liver fibrosis.

## Materials and Methods

### Liver Fibrosis Model and Chlorophyllin Treatment

All animal experimental procedures in this study complied with guidelines as outlined in the “Guide for the Care and Use of Laboratory Animals” (National Research Council, United States). The animal protocols were approved by the Institutional Animal Care and Use Committee, the College of Life Sciences, Sichuan University. Briefly, 4–5 week old BALB/c male mice (Beijing HFK Bioscience) were maintained in a controlled environment (12:12 light-dark cycle) with free access to both food and water. After 2 weeks of adaptation, the mice were randomly divided into three groups: control, fibrosis, fibrosis treated with chlorophyllin (*n* = 5–7 per group); and the experiment was repeated. The liver fibrosis of mice was induced via intraperitoneal injections of carbon tetrachloride (CCl_4_, twice per week in a progressive regimen: 0.5 μL/g body weight for the first two treatments, then 1.5 μL/g for the next two treatments, and 2.0 μL/g for additional 6 weeks) for total 8 weeks. One group of mice was treated with same volume of mineral oil as control. One group of fibrotic mice was treated by chlorophyllin (Chengdu Tongdei Pharmaceuticals, China) in the drinking water at a dose of 25 μg/mL. Actual volume of consumption was recorded. Averagely, mice drink 3–5 mL per day. Pure drinking water was applied for the control and fibrosis groups. According to our daily observation, the green pigment did not cause any sign of abnormality or sickness. At the end of experiment, the mice were sacrifice through cervical dislocation. The liver, ileum tissues, and fecal pellets were collected and stored at -80°C for analysis.

### Short-Term Oral Administration of Chlorophyllin on Intestinal Microflora of the Mice With Liver Fibrosis

BALB/c male mice under liver fibrotic induction by CCl_4_ treatment for 4 weeks were randomly divided into two groups and received (1) low dose (5 μg/g body weight), or (2) high dose chlorophyllin (25 μg/g). Fresh fecal pellets were collected at 0, 2, 4, and 8 h. for microflora analysis (*n* = 2–4 for each condition).

### Plasma Lipopolysaccharide (LPS) and Cytokine Measurements

he plasma LPS concentration was determined via Limulus Amebocyte Extract kit (Chinese Horseshoe Crab Reagent Manufactory, Xiamen, China). Plasma was diluted in the processing buffer into 1/10, and heated for 10 min at 70°C. Then the plasma LPS content was analyzed following the manufacturer’s instruction. Plasma TNF-α concentration was analyzed using ELISA kit (Mercodia, Uppsala, Sweden).

### Histological Analysis

Liver tissues were fixed in 4% paraformaldehyde, and Hematoxylin, and Eosin (H&E) staining was used for histologic analysis. Liver fibrosis was determined through Masson’s Trichrome staining. and the sizes of fibrotic septa were quantitated via densitometry. Lymphocyte infiltration in the liver was determined via anti-CD3 (Abcam, Cat. 16669, United States). Colorimetric images for H&E and Masson’s Trichrome staining were captured via Nikon eclipse Ti-U microscope. Tight junctions in the ileum were examined using immunofluorescent staining with anti-occludin (Santa Cruz Biotechnology, Cat. SC5562, United States) and all images were captured via the Leica TCS SP5 II system.

#### Western Blot Analysis

The ileum tissues were homogenized and lysed in RadioImmuno Precipitation Assay (RIPA) buffer containing protease inhibitors. The concentration of total protein was detected by Pierce bicinchoninic acid assay (BCA) Protein Assay Kit (Thermo, United States). Equal quality of protein samples was resolved via sodium dodecyl sulfate polyacrylamide gel electrophoresis and transferred to polyvinylidene difluoride (PVDF) membranes. The membranes were blocked in 5% nonfat milk and hybridized to primary antibodies against I-kappa-B, phosphor-I-kappa-B, IKK, phosphor-IKK (Cell Signaling Technology), or GAPDH (Zen BioScience, Cat. EE0618, China), followed by incubation with horse radish peroxidase (HRP)-conjugated secondary antibodies. Images were developed via Immobilon Western Chemiluminescent substrate (Millipore, United States).

#### RT-qPCR Analysis

The liver or ileum tissue was homogenized in 1 mL Trizol and total RNA was extracted. The RNA was converted to cDNA via the Transcriptor First Strand cDNA Synthesis Kit (Roche, Cat. 04897030001, United States). The qPCR system contained 2 μL cDNA, 0.2 μL forward primer, and 0.2 μL reverse primer (200 nM), 5 μL MIX, and DEPC water was added to 10 μL. The reaction was performed with a Bio-Rad machine Cfx96. The primer sequence information is listed in Table 1. The relative mRNA expression was normalized to the expression of RPL-19.

**Table 1:** The list of RT-qPCR primers for mice.

Gene	Forward primer, 5′ > 3′	Reverse primer, 5′ > 3′
TNF-α	TGGGACAGTGACCTGGACTGT	TTCGGAAAGCCCATTTGAGT
IL-1β	TCGCTCAGGGTCACAAGAAA	CATCAGAGGCAAGGAGGAAAAC
IL-6	CTTCCATCCAGTTGCCTTCTTG	AATTAAGCCTCCGACTTGTGAAG
Coll-1	GCTCCTCTTAGGGGCCACT	CCACGTCTCACCATTGGGG
Coll-3	GCACAGCAGTCCAACGTAGA	GCTTCTTTTCCTTGGGGTTC
TGF-β	CTTCAGCTCCACAGAGAAGA	GACAGAAGTTGGCATGGTAG
α-SMA	TCCAGCCATCTTTCATTGGGA	CCCCTGACAGGACGTTGTTA
ZO-1	ACCCGAAACTGATGCTGTGGATAG	AAATGGCCGGGCAGAACTTGTGTA
Occludin	ATGTCCGGCCGATGCTCTC	TTTGGCTGCTCTTGGGTCTGTAT
MMP-2	CAACGGTCGGGAATACAGCAG	CCAGGAAAGTGAAGGGGAAGA
MMP-9	AAACCTCCAACCTCACGGAC	CTGAAGCATCAGCAAAGCCG
MMP-13	GACCCCAACCCTAAGCATCC	CCTCGGAGACTGGTAATGGC
MMP-14	ATCTCACAGCTCGGTGTGTGTTCA	AAGGTCAGAGGGTCTTGCCTTCAA
TIMP-1	GCATGGACATTTATTCTCCACTGT	TCTCTAGGAGCCCGATCTG
TIMP-2	GCCAAAGCAGTGAGCGAGAAG	GGGGAGGAGATGTAGCAAGGG


#### Gut Microbiota Analysis

SYBR green-based qPCR analysis of 16S rRNA genes was used to quantitate the relative abundance of gut bacteria. Fecal microbe DNA was extracted using stool DNA kit (Omega, China). The qPCR system contained 2 μL DNA, 0.2 μL forward primer, 0.2 μL reverse primer (200 nM), 5 μL MIX, and DEPC water was added to 10 μL. Then, it was analyzed with the Bio-Rad Cfx96 and the value was expressed as the percentage of common bacterial readouts as internal reference (Table 2). The accuracy of the qPCR based 16 rDNA analysis was previously validated by sequencing the PCR products.

**Table 2:** The list of microbiota 16S rDNA primer.

Gene	Forward primer (5′ > 3′)	Reverse primer (5′ > 3′)
All bacteria	ACTCCTACGGGAGGCAGCAG	ATTACCGCGGCTGCTGG
Firmicutes	GGAGTATGTGGTTTAATTCGAAGCA	AGCTGACGACAACCATGCAC
Bacteroidetes	GGAGCATGTGGTTTAATTCGATGAT	AGCTGACGACAACCATGCAGG


### Impact of Chlorophyllin on the Inflammatory Signaling Pathways of Intestinal Epithelial Cells

The intestinal epithelial cells HT-29 and HCT-116, from colon cancer, were pretreated with chlorophyllin at 50 μM for 60 min prior to being challenged with endotoxin (LPS) at 0.5 μg/mL for 15 min. Nuclear and cytoplasmic protein fractionations were separated according to the manufacturer’s instructions (Beyotime Nuclear and Cytoplasmic Protein Extraction Kit, China). Briefly, about 5.0 × 10^5^ cells for each condition were prepared in cytoplasmic lysis buffer and the nuclear protein was isolated via nuclear extraction buffer. Protein concentration was measured using the Pierce BCA Protein Assay kit. The preparation of cytoplasmic and nuclear fractions followed previously described methods (Yang et al., 2017). Cytoplasmic fractions were monitored via Western blot analysis for GAPDH and beta-actin and nuclear fractions were confirmed via histone H3 and nuclear lamin A/C. In such frames, nuclear translocation of Nuclear factor κB (NF κB)-p65 and NFκ B-p50 were measured.

#### Statistical Analysis

The data was analyzed with the software of GraphPad Prism5 and the Statistical significance was determined via *T*-tests. The results are presented as Means ± SEM. Significance was assumed for a ^∗^*p* < 0.05 or a ^∗∗^*p* < 0.01.

## Results

### Liver Fibrosis Induced by CCl_4_ in Mice Was Ameliorated by Oral Administration of Chlorophyllin

**FIGURE 1:**
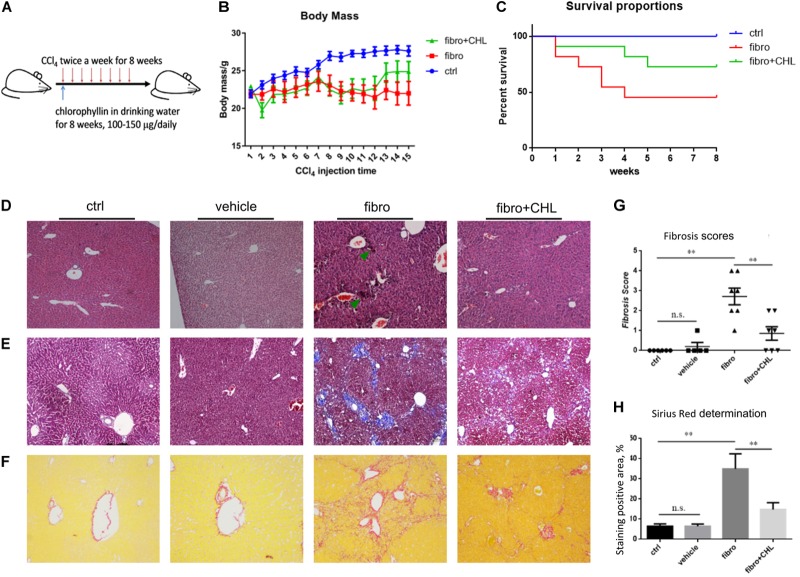
CCl_4_ induced liver fibrosis in mice is ameliorated by oral administration of chlorophyllin. BALB/c male mice were randomly divided into three groups: control, fibrosis, fibrosis treated with chlorophyllin (*n* = 6 and repeating twice). Liver fibrosis of the mice was induced via intraperitoneal injection of carbon tetrachloride (CCl_4_) for 8 weeks. Oral administration of chlorophyllin was conducted by adding to drinking water at a dose of 25 μg/mL (equivalent 5 mg/kg body mass). **(A)** Sketch of the experimental design. **(B)** Changing body mass. **(C)** Survival rate. **(D)** Hematoxylin and Eosin staining of the liver tissues. **(E)** Masson’s Trichrome staining for liver fibrosis. **(F)** Sirius Red staining for liver fibrosis. **(G)** Fibrosis scores have been determined with pathologists in a blind manner. **(H)** Semi-quantitative determination of fibrosis stained by Sirius Red. ^∗^*P* < 0.05, ^∗∗^*P* < 0.01. ^∗/∗∗^ Comparisons have been indicated with bars. Data show Means ± SEM.

BALB/c male mice were randomly divided into three groups: (1) control, (2) liver fibrosis, (3) fibrosis treated with chlorophyllin, as described in the section of Materials and Methods. Liver fibrosis of mice was induced via repeating intraperitoneal injection of carbon tetrachloride (CCl_4_) for 8 weeks. Another group of the mice under fibrotic treatment was additionally treated by chlorophyllin in the drinking water at a dose of 25 μg/mL (equivalent of 5 mg/kg body mass), Figure 1A. As shown in Figure 1B, the body mass of mice under CCl_4_ treatment decreased significantly after 7–8 regimens of toxin treatment. Chlorophyllin treatment moderately restored the body mass, but without statistical significance. About 50% of the mice under CCl_4_ treatment died over the course of the experiment, while chlorophyllin treatment reduced the mortality to 25% (Figure 1C). Histological examination via H&E staining showed large-scale of inflammation, accumulation of leukocytes, and necrosis in the parenchyma of the liver in response to CCl_4_ treatment (Figure 1D). Chlorophyllin treatment significantly reduced such necro-inflammation and liver injury in addition to improved mortality. Fibrosis developed with CCl_4_ treatment over 8 weeks, as shown with Masson’s Trichrome staining, showing fibrotic septa and partial nodulation (Figures 1E,F). In contrast, chlorophyllin treatment significantly reduced morphological liver fibrosis. We further determined the extent of fibrogenesis with a blind fibrotic scoring system and imaging analysis on the Sirius Red staining. As shown in Figures 1G,H, chlorophyllin treatment substantially reduced the liver fibrosis scores.

### Chlorophyllin Treatment Increased the MMP/TIMP Ratio, Which May Promote Fibrolysis and Resolving Liver Fibrosis

**FIGURE 2:**
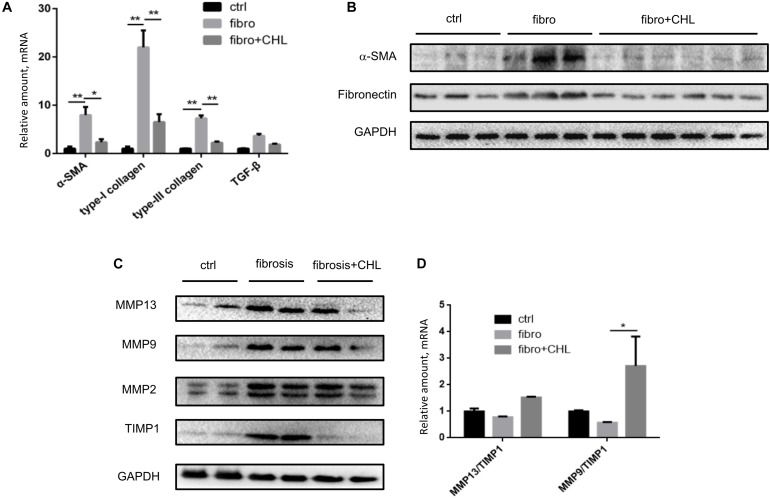
Chlorophyllin treatment increases MMP/TIMP ratio, which may promote fibrosis resolution. **(A)** Reverse transcription coupled quantitated PCR analysis (RT-qPCR) analysis was used to quantitate liver fibrosis, shown the mRNA levels of alpha-smooth actin, type-I collagen, type-III collagen, and transforming growth factor beta-1. **(B)** Western blot analysis of liver fibrosis by where alpha-smooth muscle actin and fibronectin were examined. **(C)** Western blot analysis of matrix metalloproteinases (MMP9 and MMP13) and corresponding tissue inhibitors of matrix metalloproteinases (TIMP1, TIMP2). **(D)** Ratio of MMP9/TIMP1 and MMP13/TIMP1 based on densitometry data. ^∗^*P* < 0.05, ^∗∗^*P* < 0.01. ^∗/∗∗^ Comparisons have been indicated with bars. Data show Means ± SEM.

There are two types of MMPs. One group (including MMP2 and MMP14) is constitutively expressed in most tissue and responsible for the turnover of ECM and tissue homeostasis, while the second group (including MMP9 and MMP13) is involved in tissue injury and fibrogenesis. Conversely, MMP activities are inhibited by Tissue Inhibitors of Matrix Metalloproteinases (TIMPs). As far, the net activity of ECM turnover or fibrosis regression depends on the ratio of MMPs over TIMPs. Our previous work demonstrated the critical roles of MMPs in liver injury, repair, and resolution, relying on the subtypes of the proteinases and tissue inhibitors (Han et al., 2004; Qin and Han, 2010; Lu et al., 2013). Indeed, the results of the Western blot analysis showed that the protein levels of alpha-smooth muscle actin and fibronectin were elevated in the liver tissue of mice under fibrotic treatment. However, the oral administration of chlorophyllin could fully down regulate these major fibrotic proteins. Liver fibrosis was also quantitated via RT-qPCR analysis (Figure 2A). As shown in Figure 2A, the transcription levels of alpha-smooth muscle actin, type-I collagen, type-III collagen, and transforming growth factor beta-1 were significantly up-regulated in response to CCl_4_ treatment for 8 weeks, which is in line with the morphological evidence of liver fibrogenesis. In contrast, chlorophyllin treatment ameliorated these transcriptional parameters of liver fibrosis, also in agreement with the readouts of morphological fibrosis. The hepatic protein levels of alpha-smooth actin and fibronectin were increased in the course of liver fibrosis (Figure 2B). And oral administration of the green pigment, chlorophyllin, could partially suppress the expression of these fibrotic proteins in the liver. The protein levels of MMP9 and MMP13 were increased in the fibrotic liver, indicating dynamic turnover of fibrotic ECM in the course of fibrogenesis (Figure 2C). However, chlorophyllin did not impact on these two MMPs. Importantly, TIMP1, the tissue inhibitor for MMP9/MMP13 (injury type MMPs) was significantly down regulated by chlorophyllin treatment, while TIMP2, the tissue inhibitor for MMP2/MMP14 (constitutive MMPs) was not impacted by administration of chlorophyllin. The ratio of MMP9/TIMP1 and MMP13/TIMP1 showed increments in the liver after chlorophyllin treatment, indicating that administration of chlorophyllin may promote the fibrolysis and resolving liver fibrosis (Figure 2D). Taken together, these results from multi-parameter analysis indicate that oral administration of chlorophyllin attenuated liver fibrosis, likely via elevation of the MMP/TIMP ratio, which consequently promotes the resolution of liver fibrosis, as demonstrated in the animal model.

### Liver Inflammation Is Attenuated by Oral Administration of Chlorophyllin

**FIGURE 3:**
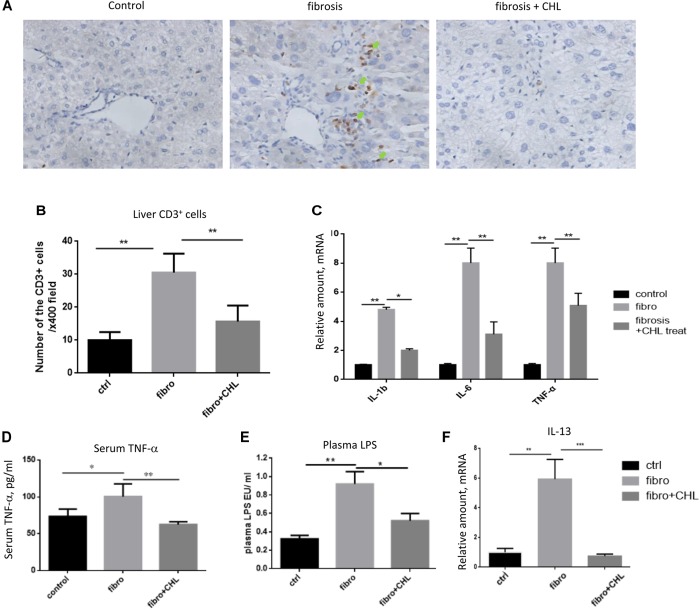
CCl_4_ induced liver inflammation is attenuated by oral administration of chlorophyllin. **(A,B)** CD3+ lymphocytes were measured via immunohistochemical staining. The CD3+ cells are indicated by green arrows. **(C)** Expression of inflammatory cytokines including IL-1beta, IL-6, and TNF-alpha detected via RT-qPCR analysis. **(D)** Systemic inflammation as the plasma TNF-alpha levels was determined via ELISA analysis. **(E)** Plasma endotoxin levels are measured by Limulus Amebocyte Extract kit. **(F)** Expression of interleukin-13 was detected by RT-qPCR analysis. ^∗^*P* < 0.05, ^∗∗^*P* < 0.01. ^∗/∗∗^ Comparisons have been indicated with bars. Data show Means ± SEM.

Hepatic fibrosis, as a protective measure and wound-healing program, is driven by local and systemic inflammation, featured by cytokines and growth factors. As shown in Figures 3A,B, infiltration of CD3+ lymphocytes was evident and accumulated around the central vein of the fibrotic liver. Oral administration of chlorophyllin reduced the infiltration of the T cells in the parenchyma of the liver inflammatory lesion. Accordingly, expression of inflammatory cytokines including interlukin-1beta, interleukin-6, and tumor necrosis factor-alpha was increased in the fibrotic liver, but it was suppressed by chlorophyllin (Figure 3C). Systemic inflammation as determined by serum TNF-alpha levels was also elevated in fibrotic mice but it was down regulated by chlorophyllin treatment (Figure 3D). Plasma endotoxin levels were measured by serum LPS, which indicated that gut bacterial translocation was suppressed by chlorophyllin treatment (Figure 3E). Bacterial translocation in combination with microbial toxin and immunogenic components, through activation of pattern recognition receptors (PRR), are known to facilitate liver fibrosis (Neugebauer et al., 2008; Zhang et al., 2016; Shi et al., 2017).

We showed recently that the hepatic macrophages undergo drastic transition from M1 phenotypes to M2 phenotypes along with liver fibrosis (Bai et al., 2017). Here, we confirmed the notion and found that oral administration of chlorophyllin could effectively down regulate the expression of IL-13, a major cytokine and marker to M2 macrophages (Figure 3F). Thus, these results imply that chlorophyllin exerted efforts to improve liver fibrosis is likely mediated by a reduction of systemic and hepatic inflammation, the driving force for tissue fibrosis.

### Impairment of the Small Intestine in Liver Fibrotic Mice Is Improved by Oral Administration of Chlorophyllin

**FIGURE 4:**
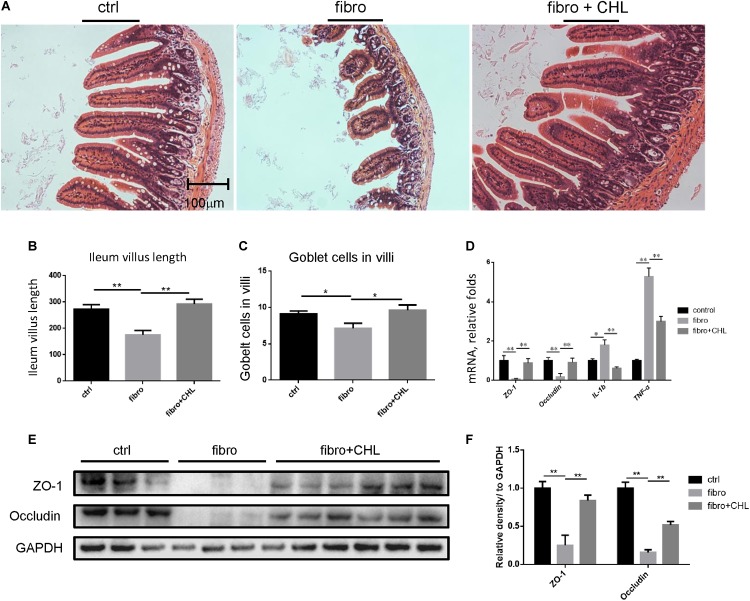
Impairment of small intestine by the liver fibrotic mice is improved by oral administration of chlorophyllin. **(A)** Histological examination via H&E staining of the distal region of small intestine. **(B)** The length of villi in the ileum was determined via Photoshop analysis based on H&E staining. **(C)** The Goblet cell numbers in the villi of the distal region of the small intestine were determined by counting in 5 views under microscopy. **(D)** The tight junction proteins including ZO1 and occludin, and pro inflammatory cytokines such as IL-1beta and TNF-alpha in the ileum were measured via RT-qPCR analysis. **(E)** Proteins of ZO1 and occludin in the distal region of the small intestine were detected via Western blot analysis. **(F)** The relative amount of tight junction proteins was semi-quantitated via densitometry analysis. ^∗^*P* < 0.05, ^∗∗^*P* < 0.01. ^∗/∗∗^ Comparisons have been indicated with bars. Data show Means ± SEM.

Increased bacterial translocation as indicated by endotoxemia and systemic TNF-alpha indicated either impairment or damage of the small intestine over the course of liver fibrogenesis. Histological examination via H&E staining showed the impairment of small intestine at the distal region, namely the ileum, of mice with liver fibrogenesis (Figure 4A). The length of microvilli was noticeably shortened and the muscular externa was thinner in the ileum of liver fibrotic mice (Figure 4B). The Goblet cell numbers in the villi were decreased in the ileum from liver fibrotic mice (Figure 4C). In contrast, oral administration of chlorophyllin ameliorated this damage of the small intestine. Moreover, the mRNA levels of tight junction proteins such as ZO1 and occludin were down regulated in the ileum of the liver fibrotic mice (Figure 4D). Oral administration of chlorophyllin partially restored the expression of tight junction proteins (Figures 4E,F). Chronic intestinal inflammation could damage tight junctions of the enterocytes which leads to bacterial translocation (Schulzke et al., 2009). As expected, the mRNA levels of interleukin-1beta and TNF-alpha were increased in the ileum of the liver fibrotic mice, indicating elevation of inflammation, but were reduced through oral administration of chlorophyllin. Western blot analysis also showed down-regulation of the proteins of ZO1 and occludin in the distal region of the small intestine in mice undergoing fibrogenesis. Importantly, oral administration of chlorophyllin restored these tight junction proteins in the ileal tissues. Taken together, these results demonstrate that oral administration of chlorophyllin could attenuate local inflammation and restores tight junctions and integrity of the small intestine, which offers a sufficient explanation for the observed reduction of endotoxemia in liver fibrosis.

### Exposure of Intestinal Epithelial Cells With Chlorophyllin Can Attenuate Inflammatory Signaling Pathways

**FIGURE 5:**
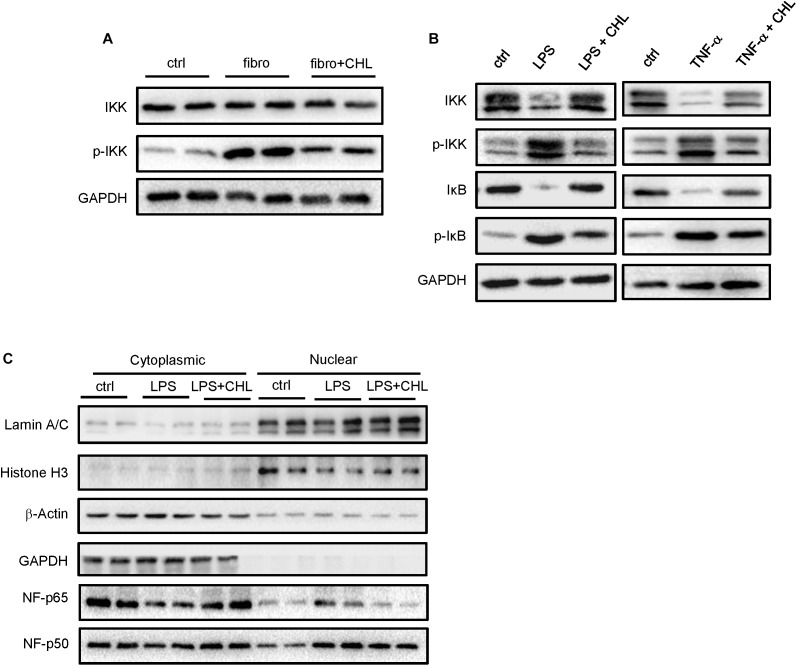
Exposure of intestinal epithelial cells with chlorophyllin can attenuate inflammatory signaling pathways. **(A)** Western blot analysis for IKK and its phosphorylation by the distal region of small intestine of the mice as described in Figure 1. **(B)** *In vitro* experiment, where intestinal epithelial cells (HCT-116) were pretreated with chlorophyllin at 50 μM for 60 min prior to challenge with endotoxin (LPS) or TNF-alpha for 15 min. Western blot analysis for the proteins of IKK, phosphor-IKK, I-kappa B, and phosphor-I-kappa B. **(C)** Cellular fractionation to measure nuclear localization for NF-kappa B/p65. The proteins in the nuclei were marked by histone H3 and nuclear matrix, lamin A/C, while cytoplasmic proteins are indicated via GAPDH. The data pooled two repeating experiment.

We speculated that the chlorophyllin treatment might directly impact on the intestinal epithelial cells to attenuate intestinal inflammation in the CCL_4_ induced fibrotic mice. Inflammatory cytokines such as TNF-alpha and interleukin-1 share common pathways including IKK and JNK cascades. Here, we examined the distal regions of the small intestine from the mices for IKK expression and activation. The results showed that the phosphorylation of IKK was significantly increased in the ileum of fibrotic mice, and the oral administration of chlorophyllin could partially suppress the IKK activation in the ileal tissue (Figure 5A). Intestinal epithelial cells (HCT-116) were pretreated with chlorophyllin at 50 μM for 60 min prior to the challenge with endotoxin (LPS) or TNF-alpha for 15 min (Figure 5B). As shown, challenging the intestinal cells with LPS or TNF-alpha could activate IKK, as indicated via increased phosphorylation of the kinases. The downstream target, I-kappa B, was phosphorylated, followed by the proteasome-mediated degradation. Treatment with chlorophyllin attenuated the IKK/I-kappa B pathway, indicating that the targeting is likely upstream of the pathway. Since LPS and TNF-alpha bind to different receptors, chlorophyllin can unequivocally block their activation, indicating a common target in the membrane complex. We then obtained the cellular fractionation to measure the nuclear localization for NF-kappa B/p65. Using HT-29 cells, nuclear protein was marked via histone H3 and the nuclear matrix (lamin A/C), while cytoplasmic proteins were indicated via GAPDH. As shown in Figure 5C, a portion of p65 NF-kappa B, in addition to its p50 partner, were translocated into the nuclear compartment in response to LPS stimulation. Chlorophyllin treatment partially suppressed the activation of the NF-kappa B pathway. We then tested the anti-inflammatory effect of chlorophyllin on hepatocyte. As shown in Supplementary Figure 1, chlorophyllin treatment suppressed the LPS-induced upregulation of inflammatory cytokine (interleukin-1 beta and interleukin-6) expression on dose-dependent pattern in HepG2 cell line.

### Dysbiosis Occurring in Liver Fibrosis Can Be Rebalanced by Oral Administration of Chlorophyllin for Eubiosis

**FIGURE 6:**
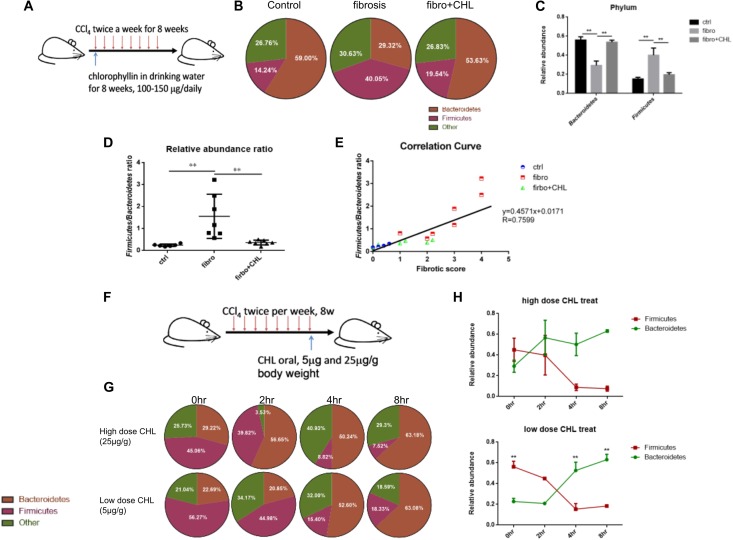
Dysbiosis occurring in the liver fibrosis can be rebalanced by oral administration of chlorophyllin for eubiosis. **(A)** Sketch of the experimental design. **(B–E)** The fecal microbiome of mice as described in Figure 1 was measured via 16S rDNA-qPCR analysis. Relative abundance of Firmicutes and Bacteroidetes in the three conditions was shown and related to endotoxin in plasma, consequently reduced hepatic inflammation and fibrogenesis. **(F–H)** Acute impact of oral administration of chlorophyllin on the gut microbiota. Liver fibrotic mice received two doses of chlorophyllin through oral gavage, at 5 and 25 μg/g, respectively. Feces were collected at the indicated time points for 16S rDNA-qPCR analysis. ^∗^*P* < 0.05, ^∗∗^*P* < 0.01. ^∗/∗∗^ Comparisons have been indicated with bars. Data show Means ± SEM.

Dysbiosis contributes greatly to bacterial translocation and endotoxemia in liver fibrosis (Gomez-Hurtado et al., 2011; De Minicis et al., 2014; Giannelli et al., 2014). As shown in Figures 6A–E, for the control mice, the phylum of Bacteroidetes, which consists of Gram-negative bacteria, were dominant in the fecal microbes. About 59% of gut bacteria beyond to Bacteroidetes, while 14% were Firmicutes. In contrast, in the fibrotic mice, the population was drastically altered, showing loss of Bacteroidetes (29% of the total) and gain of Firmicutes (40% of the total). Such dysbiosis, showing down regulation of Bacteroidetes and gain of Firmicutes, resembles in many ways to our work on NASH animal models, where liver fibrosis was evidently associated with a loss of Bacteroidetes and increased endotoxemia by the mice under high fat diet feeding (Su et al., 2016). Importantly, administration of chlorophyllin restored Bacteroidetes, which sufficiently explains the reduction of endotoxin in the plasma and consequently reduced both hepatic inflammation and fibrogenesis. A linear regression analysis for the association of dysbiosis and liver fibrosis scores showed a clear positive association between the scores of liver fibrosis and dysbiosis (elevation of Firmicutes). The changing gut microbiota through long-term treatment may derive from direct impact of chlorophyllin on the microbes or via the host innate immunity such as secretion of anti-microbial peptides. For such regard, we tested if chlorophyllin could directly impact on the gut microbiota in an acute manner. The liver fibrotic mice were given two doses of chlorophyllin by oral administration, and the gut microbiota was measured. As shown in Figures 6F–H, after oral administration for 2–4 h, the Firmicutes in fecal samples were significantly reduced, while the abundance of Bacteroidetes was restored, indicating that chlorophyllin might directly impact gut microbiota.

## Discussion

In this study, we show that the green-plant pigment in the form of chlorophyllin can ameliorate liver fibrosis. We furthermore explored the underlying potential mechanism. Liver fibrosis, or cirrhosis, is abnormal wound healing. Instead of restoration of the tissue structures with properly organized epithelia and stroma texture, fibrosis generates fibroplasia, featured by an accumulation of stiff fibrotic ECM and contractile myofibroblasts. Independent of the actual causes, either microbial infection or toxin exposure (alcohol abuse and xenobiotics), activation or trans-differentiation of hepatic fibrosis is the basis of the fibrogenesis. Wound healing is driven by growth factors such as TGF-beta, which are stored as latent forms in the ECM. MMPs produced by stellate cells are responsible for the release of growth factors as previously demonstrated by animal models (Lu et al., 2013). Induction and maturation of MMPs relies on inflammatory signals such as IL-1 and TNF-alpha from leukocytes through inflammatory pathways (Han et al., 2001, 2004). It has been reported that bacterial translocation and endotoxemia from gut are critical for systemic and hepatic inflammation (Zhang et al., 2010). This has been demonstrated by our recent work, showing that oral administration of cationic resin can deplete gut endotoxin and relieve hepatic inflammation, steatosis, and fibrosis (Zhu et al., 2017). Bacteroidetes and Firmicutes are the two major phyla of the gut microbiome; the former consist of Gram-negative bacteria. Thus, it is likely that death of Bacteroidetes (showing as reduced abundance in the gut microbiota) may contribute to bacterial translocation, endotoxemia, systemic and hepatic inflammation, and liver fibrogenesis. However, the integrity of small intestine, including its innate immunity, anti-microbe peptides, mucosa, and tight junctions and the consequent gut eubiosis are two critical factors for host defense and metabolic homeostasis.

Chlorophyllin is a water-soluble salt that is semi-synthesized from chlorophyll. Its most common form is a sodium/copper derivative that can be used as a food additive and is used in alternative medicine. It is also widely used as a food-coloring agent. Through its flat chlorin ring, chlorophyllin is able to bind to environmental mutagens such as polycyclic aromatic hydrocarbons (Breinholt et al., 1995; Blum et al., 2001). In an animal model, dietary supplementation of chlorophyllin at 4 mg/kg body weight inhibited the development of MNNG-induced forestomach carcinomas via down-regulation of the expression of TGF-beta RI, TGFbeta RII, and Smad 2 and 4 and up-regulation of Smad 7, thus abrogating canonical TGF-beta signaling (Thiyagarajan et al., 2014). A further study showed that dietary chlorophyllin can abrogate 7,12-dimethylbenz-anthracene (DMBA)-induced hamster buccal pouch (HBP) carcinogenesis; furthermore, the authors showed that Wnt/beta-catenin and VEGF signaling are suppressed by chlorophyllin (Nagini et al., 2012).

In this study we found that chlorophyllin is able to ameliorate the hepatic toxin induced liver fibrosis. Mechanistically, chlorophyllin may work on two levels. First, we found that chlorophyllin can directly impact intestinal epithelial cells and suppress inflammatory signals that are initiated by LPS and TNF-alpha. The targeting action could work in the signaling pathways, since chlorophyllin can attenuate IKK phosphorylation and the consequent I kappa-B phosphorylation and degradation and ultimately, the nuclear translocation of p65. Whether chlorophyllin interacts with the receptors of the inflammatory ligand remains unknown and should be addressed in further studies. Another possibility is the interaction with plasma membrane through its flat chlorin ring. Secondly, we found that chlorophyllin can directly impact the gut microbiota. In particular, administration of chlorophyllin can promptly restore eubiosis, showing restoration of Bacteroidetes and reduction of Firmicutes. Such a finding is important because it can also explain the observed reduction of plasma endotoxin, preassembling through the prevention of the death of Bacteroidetes, the Gram-negative bacteria that may contribute to the plasma endotoxin via intestinal-hepatic circulation. Our recently published study reported that administration of cationic resin (cholestyramine) could sufficiently attenuate liver fibrosis induced by high-fat diet along with vitamin D deficiency (Zhu et al., 2017). In such a scenario, chlorophyllin may work as a prebiotic that can modulate gut microbiota. Whether chlorophyllin can directly suppress Firmicutes in the gut remains a subject under investigation. LPS binding induces dimerization of the TLR4–MD-2 complex, which is proposed to enable dimerization of the intracellular TIR domains and recruitment of adaptor molecules such as MyD88. We speculated the flat ring structure of chlorophyllin might directly insert into endotoxin and impair the binding to TLR4 on the membrane. For this regard, using Isothermal Titration Calorimetry (ITC) we measured the disassociation constant (Kd) between chlorophyllin and LPS,but the results were inconclusive and further study is needed to address the mechanism of chlorophyllin in the cells.

## Author Contributions

HZ, YY, PW, YL, ZC, and LZ performed the experiments. ML, MH, and HW analyzed the data. YaL, YZ, YoL, JZ, and MF helped in writing the manuscript and discussion. ZH, RH, and Y-PH prepared the figures and drafted the manuscript. YZ and SP edited and revised the manuscript. D-XL participated in the discussion of this study.

## Conflict of Interest Statement

YaL was employed by Chengdu Tongde Pharmaceutical Ltd., Chengdu, China. The remaining authors declare that the research was conducted in the absence of any commercial or financial relationships that could be construed as a potential conflict of interest.

## Supplementary Material

FIGURE S1 Chlorophyllin treatment suppressed the LPS-induced upregulation of inflammatory cytokine expression on dose-dependent pattern in HepG2.

## Abbreviations

CCl_4_: carbon tetrachloride
CHL: Chlorophyllin
HSCs: hepatic stellate cells
LPS: lipopolysaccharides
TNF-alpha: tumor necrosis factor-alpha
