# Supplementary figures and images for: Chlorophyllin Modulates Gut Microbiota and Inhibits Intestinal Inflammation to Ameliorate Hepatic Fibrosis in Mice
Source: Front Physiol. 2018 Dec 4;9:1671. doi: 10.3389/fphys.2018.01671 (PMC6288434; doi:10.3389/fphys.2018.01671)

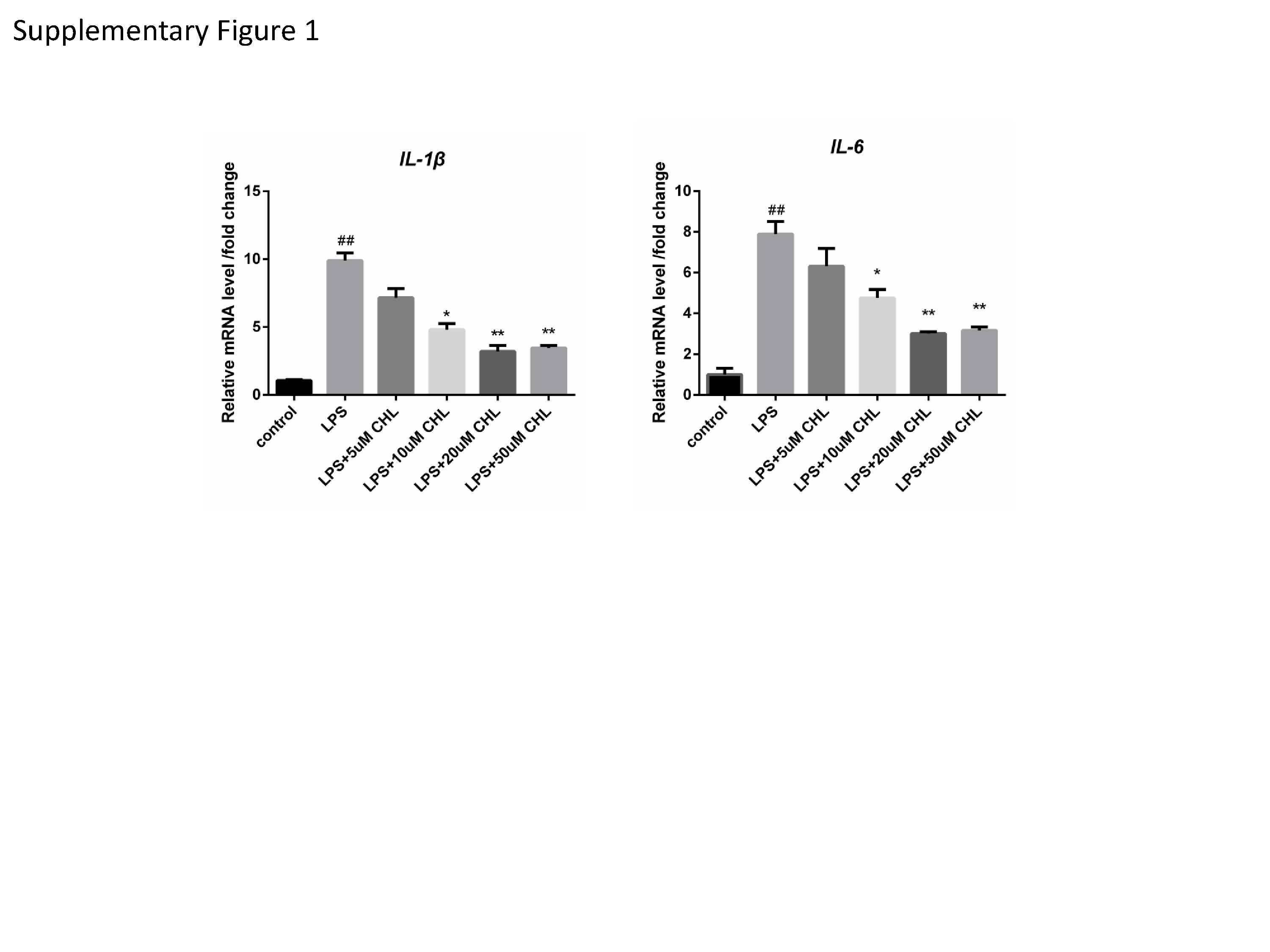

Supplement: FIGURE S1 — Chlorophyllin treatment suppressed the LPS-induced upregulation of inflammatory cytokine expression on dose-dependent pattern in HepG2. [file Image_1.TIF]
